# Fast Identification of Soybean Seed Varieties Using Laser-Induced Breakdown Spectroscopy Combined With Convolutional Neural Network

**DOI:** 10.3389/fpls.2021.714557

**Published:** 2021-10-06

**Authors:** Xiaolong Li, Zhenni He, Fei Liu, Rongqin Chen

**Affiliations:** ^1^College of Biosystems Engineering and Food Science, Zhejiang University, Hangzhou, China; ^2^Huanan Industrial Technology Research Institute of Zhejiang University, Guangzhou, China

**Keywords:** soybean seed, variety identification, laser-induced breakdown spectroscopy, convolutional neural network, voting strategy

## Abstract

Soybean seed purity is a critical factor in agricultural products, standardization of seed quality, and food processing. In this study, laser-induced breakdown spectroscopy (LIBS) as an effective technology was successfully used to identify ten varieties of soybean seeds. We improved the traditional sample preparation scheme for LIBS. Instead of grinding and squashing, we propose a time-efficient method by pressing soybean seeds into rubber sand filled with culture plates through a ruler to ensure a relatively uniform surface height. In our experimental scheme, three LIBS spectra were finally collected for each soybean seed. A majority vote based on three spectra was applied as the final decision judging the attribution of a single soybean seed. The results showed that the support vector machine (SVM) obtained the optimal identification accuracy of 90% in the prediction set. In addition, PCA-ResNet (propagation coefficient adaptive ResNet) and PCSA-ResNet (propagation coefficient synchronous adaptive ResNet) were designed based on typical ResNet structure by changing the way of self-adaption of propagation coefficients. Combined with a new form of input data called spectral matrix, PCSA-ResNet obtained the optimal performance with the discriminate accuracy of 91.75% in the prediction set. T-distributed stochastic neighbor embedding (t-SNE) was used to visualize the clustering process of the extracted features by PCSA-ResNet. For the interpretation of the good performance of PCSA-ResNet coupled with the spectral matrix, saliency maps were further applied to visually show the pixel positions of the spectral matrix that had a significant influence on the discrimination results, indicating that the content and proportion of elements in soybean seeds could reflect the variety differences.

## Introduction

Soybean is one of the most important agricultural products, which has abundant vegetable protein and oil. The yield and quality of soybeans are directly related to their variety with different genetic purity, physical purity, germination ability, and vigor (John et al., 2016; Mccarville et al., 2017; Zhu et al., 2019a). Mixed and adulterated soybean seeds cause substantial problems for farmers and lead to seed market complexities (Liu et al., 2016). With the increasing requirements for food quality, it is necessary to process different products according to different seed varieties. For instance, the soymilk and tofu made from high-protein soybeans are more delicious (Sato et al., 2014; Yu et al., 2014). Therefore, rapid identification of soybean seed varieties plays an essential role in agricultural products, standardization of seed quality, and food processing. It becomes more and more crucial to build a general discriminant model for distinguishing different soybean seed varieties with large amounts but little difference (Luo et al., 2019).

DNA analysis and protein-based technologies are regarded as powerful tools for specific and precise identification of soybean seed varieties, such as polymerase chain reaction (PCR) (Grohmann et al., 2017), high-performance liquid chromatography (HPLC) (Cho et al., 2013; Kim et al., 2013) and simple sequence repeat (SSR) analysis (Zhang et al., 2014). These genetic methods often require environmentally unfriendly chemical agents to show results. By comparison, the spectroscopy technique does not need any chemical agent and causes minor damage to samples. Therefore, the spectroscopy technique can be an alternative as a non-genetic method to achieve fast genotype discrimination.

Laser-induced breakdown spectroscopy (LIBS) is an atomic emission spectroscopy technique which is characteristically fast, micro-damaging, and with simple sample pretreatment (Erler et al., 2020). In a typical LIBS system, a high-energy pulsed laser is transmitted nearly to the surface of the sample. After that, the plasma is created with the vaporization and excitation of the sample (Li et al., 2019). The emitted spectra from the plasma are collected for multi-element analysis (Liu et al., 2019; Wang et al., 2020). So far, LIBS technology has been widely used in qualitative and quantitative analysis in agricultural products such as rice (Luo et al., 2020), psoralea corylifolia seeds (Dhar et al., 2013), cucurbit seeds (Singh et al., 2017), coffee beans (Song et al., 2017), soybean seeds (Gamela et al., 2020; Larios et al., 2020), and grape seeds (He et al., 2020). However, the samples above were grounded and pressed into tablets before collecting LIBS spectra for better signals, which greatly reduced the detection efficiency. This study proposed an innovative method of pressing soybean seeds into a culture plate filled with rubber sand, with a ruler used to ensure a relatively uniform surface in height. Then the soybean seeds could be directly shot by laser beam without any specific pretreatments, markedly reducing the time cost. The LIBS spectra of 2,000 soybeans contributed to establishing a discriminant model with improved generalization due to extensive data.

Much attention has been paid to the traditional machine learning algorithms for modeling LIBS data but little to deep learning and its interpretation (Zhao et al., 2019). For example, support vector machine (SVM) is a commonly used algorithm in machine learning (Liakos et al., 2018). SVM is intrinsically a binary classifier that constructs a linear separating hyperplane to classify data instances (Vapnik and Chapelle, 2000). On account of kernel trick and structural risk minimization principles, SVM usually presents a better performance in classification and regression (Hesami et al., 2020). It has been applied in various fields in agriculture (Ang and Seng, 2021), such as plant breeding (Yoosefzadeh-Najafabadi et al., 2021), pest detection (Ebrahimi et al., 2017), and soil condition prediction (Morellos et al., 2016). However, SVM usually takes a long time to search for optimal parameters. What is more, for multiclass classification, SVM may have a lower classification accuracy than artificial neural network (ANN) (Xia et al., 2018). Therefore, it is necessary to use advanced methods like deep learning. Convolutional neural network (CNN) is one kind of deep learning, which is often used for image and speech recognition. CNN can also be used for spectral data processing (Yan et al., 2021). Compared to ANN, CNN is more likely to reduce the risk of overfitting by sharing the same convolution parameters. Moreover, CNN can identify important spectra regions by applying the same convolutional kernel in a spectrum (Acquarelli et al., 2017). A saliency map is a powerful tool to show the important regions visually (Peruzzi et al., 2021). As we all know, CNN is particularly suitable for image data processing relying on the two-dimensional and self-adaptive characteristics of the convolution kernel. Therefore, we proposed a new form of input data by connecting three spectra of a soybean seed into a spectral matrix. Because of the self-adaptive characteristic of convolution kernel, we expect CNN to learn the important features of the spectral matrix and further improve the modeling effect. At the same time, we can use t-distributed stochastic neighbor embedding (t-SNE) (van der Maaten and Hinton, 2008) to display the learning effect for different layers in CNN. Thus, the main objectives of this study are: (1) to compare identification accuracy between machine learning and deep learning; (2) to use spectral matrix as the input of CNN; (3) to use t-SNE to visually observe the learning process of CNN; (4) to use saliency maps to find the more influential positions in the spectral matrix on the discriminant results.

## Materials and Methods

### Sample Preparation

**FIGURE 1:**
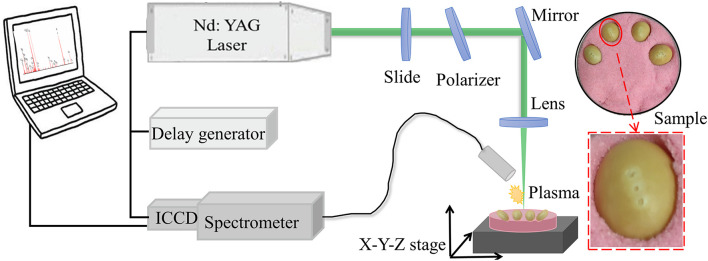
The schematic diagram of the laser-induced breakdown spectroscopy (LIBS) experiment.

The soybean seeds from a single batch were purchased from a seed company in Shuyang Pengyuan horticulture farm, Suqian, Jiangsu, including Guandou 1, Zhoudou 23, Hedou 13, Jiadou 23, Hedou 33, Lvbaoshi, Hedou 25, Qihuang 34, Zhonghuang 13, and Wandou 15, which were correspondingly numbered from variety 1 to variety 10 for convenient description. Two hundred seeds free from damage and disease spots for each variety were selected. Then, without any other pretreatment, every four soybean seeds were pressed into rubber sand in a culture plate using a ruler to ensure a relatively uniform surface height (Figure 1) for the LIBS experiment.

### Experimental Setup

The experiment was completed using the LIBS system as shown in Figure 1. A Q-switched Nd:YAG pulse laser (Vlite-200, Beamtech Optronics, Beijing, China) was used to generate a pulse laser at 532 nm with a pulse duration of 8 ns, beam diameter of 7 mm, and maximum energy of 200 mJ. Then, the pulse laser was guided to the sample by an optical system, in which a glass slide and a polarizer was combined to control laser energy and a plano-convex lens (*f* = 100 mm) was fixed to focus the laser beam 2 mm below the surface of the sample. To avoid repeat ablations, an X-Y-Z motorized stage was applied to move the sample every ten accumulation shots. The LIBS spectra were separated by the high-resolution Echelle spectrometer (ME5000, Andor Information Technology Ltd., Belfast, United Kingdom) in the range of 230–904 nm with 0.01 nm resolution and then collected by an intensified charge coupled device (ICCD) camera (DH334, Andor Information Technology Ltd., Belfast, United Kingdom). The delay generator (DG645, Stanford Research Systems Inc., Sunnyvale, CA, United States) was applied to adjust the delay time between the action of the laser ablation and the camera working.

The pulse energy, delay, and integration time were the three important parameters for LIBS, which were optimized as 60 mJ, 2 μs, and 10 μs, respectively, improving the data quality. In the air environment, three different points on a soybean seed were used to be ablated as shown in Figure 1. The horizontal distance between two adjacent points is one millimeter and the middle point is the highest. At each point, the spectra with 10 times accumulation were collected to gather information from the surface to the inside of the soybean seed. The average spectra were taken as the final spectrum. Thus, one soybean seed produced three spectra and a total of 6,000 spectra were produced in this experiment. It only took 30 s to complete the spectral acquisition for one soybean seed.

### Data Preprocess

LIBS spectra within soybean seeds contained obvious random noise in the head and end of the spectra. Thus, the wavelengths in the range of 242–882 nm were studied. To reduce fluctuations from point to point, area normalization method was used for each LIBS spectrum following the equation below:


(1) $$X_{i}=\frac{x_{i}}{\sum_{i=1}^{n}x_{i}}$$

where *x_i_* is the *i*th variable relative intensity measured by LIBS system, *n* is the total number of LIBS spectral variables, *X_i_* is the relative intensity by area normalization. Then variables with near-zero standard deviation were removed to reduce the dimension of LIBS spectra (Boucher et al., 2015). All soybean seeds were randomly divided into the calibration set, validation set, and prediction set according to the ratio of 3:1:1. The number of the LIBS spectra in the three data sets was 3,600, 1,200, and 1,200, respectively.

### Principal Component Analysis

Principal component analysis (PCA) is a commonly used method to generate an easy visualization of the distribution of samples (Velioglu et al., 2018; Zhu et al., 2019b). The principle of PCA is to find the unit vector to maximize the variance after the original spectral data is projected on the vector, so that the information of the original spectral data can be retained to the greatest extent. The variance can be calculated by the following equation:


$$\sigma^{2}=\frac{1}{n}\,\sum {\left(x_{i}\,v\right)}^{2}$$

(2) =1n⁢∑vT⁢xiT⁢xi⁢v=vT⁢(1n⁢∑xiT⁢xi)⁢v=vT⁢C⁢v

Where *x_i_* is a LIBS spectrum, *v* is unit vector and *C* is covariance matrix of all pixel spectra. So, *v* = argmax(*v^T^* *C^v^*), subjected to *v^T^v*−1 = 0. We can use the lagrange multiplier method to solve *v*. The process is as below:


(3) $$\text{L}=v^{T}\,C\,v-\lambda \,\left(v^{T}\,v-1\right)$$

(4) $$\frac{\partial L}{\partial v}=2\,C\,v-2\,\lambda \,v=0\overset{y\,i\,e\,l\,d\,s}{⟶}C\,v=\lambda \,v$$

(5) $$\frac{\partial L}{\partial \lambda }=0\overset{y\,i\,e\,l\,d\,s}{⟶}v^{T}\,v=1$$

Where, λ is the lagrangian multiplier.

Therefore, λ is the eigenvalue of *C* and *v* is eigenvector of *C*. Through PCA, we can get different unit vectors *v*s with different λs. The larger is, the greater the contribution rate of *v* is. In this study, the first three *v*s were used to generate three principal components (PCs). *PC1* = *Xv*_1_,*PC2* = *Xv*_2_,*PC3* = *Xv*_3_. We can intuitively see the clustering of samples by scoring 3-D scatter plots of PC1, PC2, and PC3.

### Discriminant Analysis Method

#### Machine Learning

K-nearest neighbor (KNN) is the simplest classification algorithm in machine learning. The distances between samples are calculated first. Then, k nearest samples are considered to be in the same category. In this study, k is determined by the discriminant accuracy of the validation set and selected in the range of 3–20.

SVM is a stable supervised classification model, which is also suitable for small and high-dimensional data (Vapnik and Chapelle, 2000; Scholkopf et al., 2001). In the process of SVM modeling, the optimal hyperplane is searched to separate the samples by exploring support vector points. At the same time, the structural risks should be minimized. Due to the simplicity of radial basis function (RBF) and its ability to solve complex nonlinear problems, RBF was selected as the kernel function in this study. Kernel function parameter *g* determines the linearity of the hyperplane and the regularization parameter *c* determines the capacity of fault tolerance (Yu et al., 2016). In order to guarantee the better performance of SVM, the optimal parameters *c* and *g* were selected through grid-search procedure from 10^–8^ to 10^8^ and determined by classification accuracy of five-fold cross validation.

#### Deep Learning

Deep learning has become the hottest topic in the field of artificial intelligence. CNN is one of the well-known deep learning structures for classification (Kamilaris and Prenafeta-Boldu, 2018; Ren et al., 2020). In this study, three kinds of common network structures called LeNet (Lecun et al., 1998), DenseNet (Huang et al., 2017) and ResNet (He et al., 2016) were compared firstly. Then according to the discriminant results, two kinds of self-proposed network structures based on ResNet were further studied. The detailed structures based on LeNet and DenseNet are shown in Supplementary Figures 1, 2.

**FIGURE 2:**
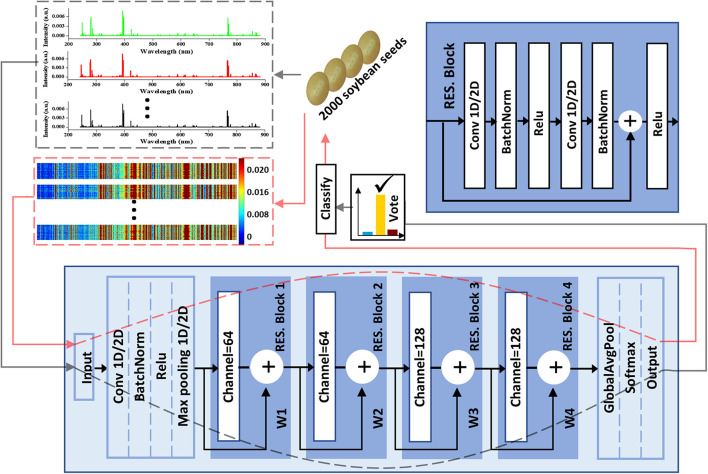
The soybean seeds classification flowchart including data input, ResNet-based classifier, and a majority vote strategy.


For three kinds of ResNets, basic network architecture is shown in Figure 2. Residual block (RES. Block) is the main characteristic that distinguishes this network structure from others. RES. Block is composed of two convolution layers (Convs), each of which is followed by a batch normalization process and rectified linear unit (Relu) activation function. The two Convs have the same parameters in kernel size, padding, and strides with values of 3, 1, and 1. For four RES. Blocks, the channel number of Convs was 64, 64, 128, and 128, respectively. It is worth noting that the input data can be propagated forward directly to the data before passing through the last layer. For common ResNet, the value of the propagation coefficient *W* is 1. We proposed the self-adaption of *W* including propagation coefficient adaptive ResNet (PCA-ResNet) and propagation coefficient synchronous adaptive ResNet (PCSA-ResNet). For PCA-ResNet, there is no limit between the four *W* and they are updated separately during the back-propagation process. For PCSA-ResNet, the four *W* are updated synchronously during the back propagation following the equation below:

(6) $$W_{1}=W_{2}=W_{3}=W_{4}=W^{*}$$

Between the input layer and the RES. Block 1, there was a pretreatment process as shown in Figure 2. In the process, the channel number, kernel_size, padding, and strides of Conv were 64, 7, 3, and 2, respectively, to deal with more information at once. A soybean seed could produce three spectra which could be treated separately or concatenated together into a spectral matrix. Therefore, all the Convs in 1D-CNN had two states including one dimension (1D) or two dimensions (2D) corresponding to different data forms. Additionally, a majority vote was employed to make the final decision for classification when the data form was the first one and the corresponding data transmission flow was marked using gray dotted lines. Another data transmission flow for the spectral matrix was marked using red dotted lines.

VGG is another common network structure for image processing (Simonyan and Zisserman, 2014). The model based on VGG was built as a comparison. The detailed structure is shown in Supplementary Figure 3. In order to compare the modeling effects of different 2D-CNNs, the same pretreatment process as shown in Figure 2 (between the input layer and the RES. Block 1) was added to 2D-LeNet, 2D-DenseNet, and 2D-VGG.

Deep learning models were trained using stochastic gradient descent (SGD) with different learning rates. At the beginning of model training, the learning rate was high and gradually decreased to approximate the optimal accuracy. For each learning rate, there was a threshold for the accuracy of the validation set, which gradually increased with the decrease of the learning rate. When the accuracy of the validation set reached the threshold, the training of the model was stopped. If the accuracy of the validation set could not reach the threshold, the model training would be stopped after 100 iterations. The learning rates and thresholds were set together at different stages of the training process of the model. Taking PCSA-ResNet based on spectral matrix as an example, the learning rates were set as 0.25, 0.124, 0.05, and 0.01, respectively, and the corresponding thresholds were set as 0.84, 0.86, 0.88, and 0.887. The accuracy of the validation set finally converged to 0.887.

### Model Evaluation and Visualization

Discriminant accuracy was used to evaluate each model in this study, defined as the ratio of the number of correctly discriminated soybean seed to the total number. To further evaluate model performance, four common evaluation indicators including precision, recall, F-measure, and Matthews correlation coefficient (MCC) were calculated. The corresponding formula refers to the article by Yoosefzadeh-Najafabadi et al. (2021). In this article, the average value of the four indicators was used for a more convenient evaluation.

A confusion matrix was applied to analyze the detailed effects of classification further. The difference between the prediction results and actual results for each soybean seed could be visually observed. The confusion matrix consists of a square matrix whose vertical axis represents the true category and horizontal axis represents the predicted category. Therefore, the number on the diagonal indicated the number of soybean seeds correctly classified.

T-SNE was used to visualize the clustering process of the extracted features from the deep learning model. It could realize the nonlinear dimension reduction of high-dimensional spectra data (Husnain et al., 2019). In t-SNE, the Gaussian distribution’s perplexity was defined as 30, and the initial dimensions of PCA were defined as 12 for layers of Max pooling and RES Block4. For Dense layer, since the length of the feature vector was 10, the dimensions of PCA were set as 6, which should be smaller than 10. The spectral matrix (similar to image data format) was first reshaped into tensor in three dimensions including channel and image, and then each image data of channels was averaged (Zhang et al., 2020).

The deep learning model could calculate the weight of each pixel on the input image (spectral matrix) through the back-propagation algorithm. The graph composed of the weight value of each pixel was called the saliency map. Through the saliency map, we could visually see the pixel positions that had a higher influence on the discrimination results. The calculation formula of the weight of each pixel was as follows:


(7) $$G\,r\,a\,d_{I}=\frac{\partial \left(D\,e\,n\,s\,e_{I}\times C\,o\,r\,r\,e\,c\,t_{I}\right)}{\partial I}$$

Where, *I* is spectral matrix, *Dense* is the output vector (ten probability values) of Dense 10, *Correct* is a vector of 0 and 1 that corresponds to the *Dense*. For example, if the spectral matrix comes from variety 3, the third position is set as 1 and the others are set as 0.*Grad* is the gradient of the spectral matrix.

### Software and Hardware

The machine learning algorithms were run on Matlab R2014b (The MathWorks, Natick, MA, United States). The software was installed on a Windows7 Desktop with Intel Xeon E5-2620 and 64 GB RAM. CNN was deployed on the framework of Apache MXNet1.4.0 in another computer of Ubuntu Desktop with GTX1080Ti (NVIDIA, California, United States) and 48 GB RAM.

## Results and Discussion

### Average Spectral Analysis

**FIGURE 3:**
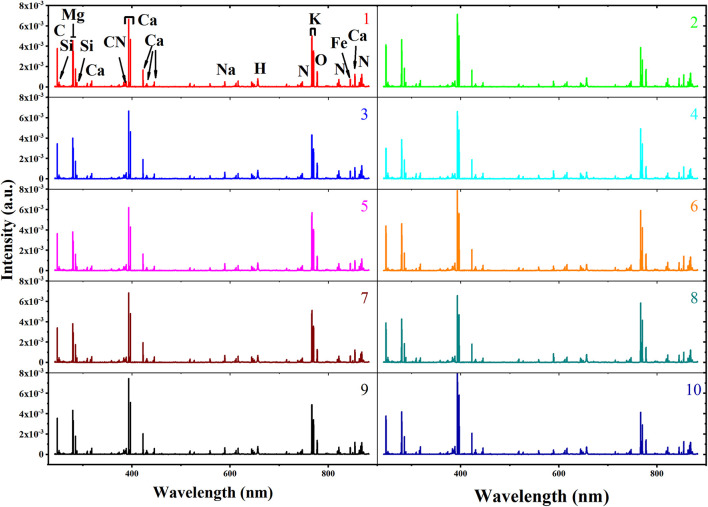
The average spectra of soybean seed samples including different varieties numbered 1–10.

The average spectra of soybean seeds from 10 varieties are shown in Figure 3. The ten LIBS spectra showed a high degree of similarity in the position of excitation peak, as they all came from the same agricultural product called soybean. Based on the National Institute of Standards and Technology (NIST), the elements corresponding to the excitation wavelength were marked in the average spectra from variety 1 (Guandou 1). The marked molecular bands CN 0–0 (around 388 nm) are usually associated with organic compounds (Fernandez-Bravo et al., 2013). It is well known that soybeans are rich in calcium, leading to many excitation lines representing calcium that could be observed at 317.93 nm, 393.37 nm, 396.8 nm, 422.67 nm, 430.25 nm, 445.48 and 854.21 nm. In addition, some microelements such as C (247.86 nm), Si (251.61 nm), Mg (279.55 nm, 280.27 nm), H (656.28 nm), K (766.49 nm, 769.90 nm), O (777.54 nm), and N (746.83 nm, 821.63 nm, and 868.03) and microelements like Fe (844.80 nm) and Na (589.59 nm) could also be easily recognized. Although the signal intensity varied among different varieties, it was difficult to distinguish them just by LIBS spectra intuitively. Thus, it was necessary to adopt mathematical data analysis to identify soybean seed varieties.

### Principal Component Analysis

**FIGURE 4:**
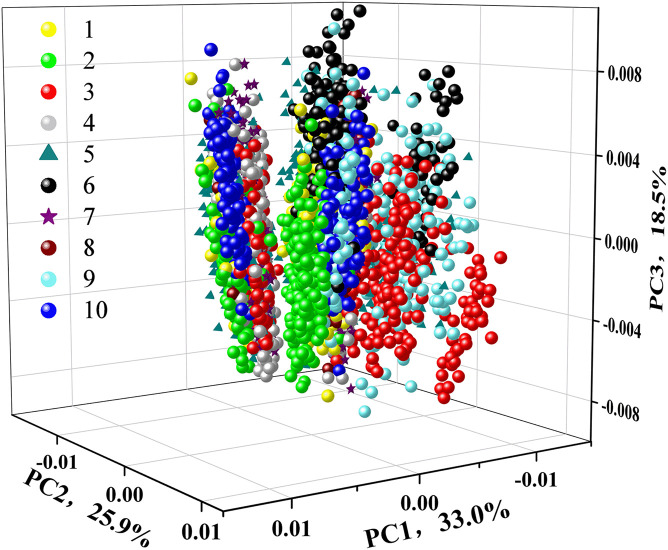
3D scatter plot of 10 different varieties of soybeans based on the first three principal components (PCs).

To test the feasibility of an unsupervised classification, a qualitative analysis of PCA was applied to explore the differences among ten different varieties of soybean seeds. The 3D score scatter plot (*X*-axis: PC1, *Y*-axis: PC2, and *Z*-axis: PC3) is presented in Figure 4. The first three PCs had explained 77.4% of the variation with PC1 of 33.0, PC2 of 25.9, and PC3 of 18.5%. Each variety of soybean seeds was marked with different color or shape for better visualization. We could see a slight distinction among different varieties. But spectra from the same variety could not be completely clustered together. For variety 10 (Wandou 15) marked with a blue circle, two clusters appeared, which indicated that PCA could not explore the variety differences very well. Therefore, supervised data processing was needed to explore the differences among the ten varieties of soybean seeds.

### Machine Learning and 1D-Convolutional Neural Network

Both 1D-LeNet and 1D-DenseNet had poor performances after trying different model parameters. The accuracy in the prediction set is about 10% for them. For machine learning and ResNets, Table 1 shows the results based on a single spectrum and a majority vote. Based on a single spectrum, KNN had the worst performance with the accuracy of 64.33% in the prediction set. SVM had a higher accuracy of 84.67%. Three different kinds of 1D-ResNets were superior to machine learning. The accuracy in the prediction set was 86.42, 86.00, and 86.83%, respectively, for 1D-ResNet, 1D-PCA-ResNet, and 1D-PCSA-ResNet. For 1D-PCA-ResNet, four different propagation coefficients of *W*_1_,*W*_2_,*W*_3_,and*W*_4_ had the value of –0.15, –1.06, 0.04, and 3.13, respectively. For 1D-PSCA-ResNet, the shared *W*^∗^ had the value of 1.52. Based on a majority vote, the discriminant accuracy of the validation set and prediction set were both improved. For machine learning, SVM obtained the highest accuracy of 90% in the prediction set. For deep learning, 1D-PCA-ResNet obtained the highest accuracy of 89.50% in the prediction set.

**TABLE 1:** The results of discriminant models based on single spectrum and a majority vote.

**Model**	**Based on single spectrum**			**Based on majority vote**		
	**Cal^a^ (%)**	**Val^b^ (%)**	**Pre^c^ (%)**	**Cal^a^ (%)**	**Val^b^ (%)**	**Pre^c^ (%)**
KNN	75.56	66.42	64.33	80.67	70.25	66.75
SVM	99.97	83.33	84.67	100.00	87.00	90.00
1D-ResNet	100.00	87.42	86.42	100.00	91.75	89.00
1D-PCA-ResNet	100.00	86.75	86.00	100.00	91.00	89.50
1D-PCSA-ResNet	100.00	86.92	86.83	100.00	90.25	89.25

*^*a,b,c*^ Cal, Val and Pre are assigned, respectively, as the discriminant accuracy of calibration set, validation set, and prediction set.*

Based on a single spectrum, the discriminant effects from deep learning were superior to machine learning, revealing the advantages and effectiveness of deep learning in spectral analysis. This is because CNN has different principles in data processing from SVM. The output of each convolutional layer of CNN is directly related to small regions of the input spectrum. Thus, CNN can identify important regions of the input spectrum (Acquarelli et al., 2017). The existing research in spectral application showed that CNN might have better performance than machine learning (Brugger et al., 2021; Chen et al., 2021). The network structure based on ResNet had higher accuracy than that based on LeNet and Densenet. Therefore, residual block played a vital role in the soybean genotype discrimination coupled with LIBS spectra. The main feature of the residual block is that the data can be propagated forward more quickly through a cross-layer data path (Moussa and Owais, 2021). However, the propagation coefficient of common ResNet was set directly as 1. We believe that propagation coefficient can also be automatically learned like convolution parameters to achieve better results. The results showed that 1D-PCSA-ResNet obtained higher accuracy for a single spectrum, which aligns with our ideas. In order to determine the variety of soybean seeds, the voting strategy was proposed. The classification accuracy for both validation and prediction sets was further improved. The results illustrated the stability and effectiveness of the voting strategy. However, the optimal classification accuracy was obtained by SVM rather than 1D-PCSA-ResNet. The reason might be that SVM was characterized by minimal structural risk (Yoosefzadeh-Najafabadi et al., 2021). In the three spectra from a soybean seed, one may be misclassified. Through majority vote, the classification accuracy could be improved.

### 2D-Convolutional Neural Network

2D-DenseNet had the same poor performances as 1D-DenseNet. The accuracy in the prediction set was about 10%. For other 2D-CNNs, Table 2 shows the results. The accuracy in the prediction set of 2D-LeNet and 2D-VGG were 85.25 and 85.75%, respectively. The model based on residual block outperformed them. 2D-ResNet had an accuracy of 89.75%. 2D-PCA-ResNet had a lower accuracy of 89.00%. The four different propagation coefficients had values of –0.15, –0.92, 0.04, and 2.43, respectively. Whereas 2D-PCSA-ResNet obtained the highest accuracy in the prediction set with the value of 91.75%. The four same propagation coefficients had the value of 1.30. For 2D-ResNet and 2D-PCSA-ResNet, the average value of four indicators including precision, recall, F-measure and MCC was calculated. For 2D-ResNet, they were 0.90, 0.90, 0.90, and 0.89, respectively. For 2D-PCSA-ResNet, they were 0.92, 0.92, 0.92, and 0.91, respectively.

**TABLE 2:** The results of 2D-CNNs based on a spectral matrix.

**Format of data**	**Model**	**Cal (%)^a^**	**Val(%)^b^**	**Pre (%)^c^**
Spectral matrix	2D-LeNet	100.00	87.75	85.25
	2D-VGG	100.00	85.50	85.75
	2D-ResNet	100.00	89.50	89.75
	2D-PCA-ResNet	100.00	90.75	89.00
	2D-PCSA-ResNet	100.00	88.75	91.75

*^*a,b,c*^ Cal, Val, and Pre are assigned, respectively, as the discriminant accuracy of calibration set, validation set, and prediction set.*

The highest accuracy was 91.75% from 2D-PCSA-ResNet, whose result was still 1.75 higher than SVM by voting strategy, demonstrating that taking spectral matrix as the input of CNN could improve the classification accuracy. One spectrum could only contain the information of one point on the soybean seed while the spectral matrix could involve more sufficient information. Moreover, the 2D convolution kernel could automatically learn the joint useful information (Zhang et al., 2020). These might be the reason for a higher accuracy. Also, all four indicators of 2D-PCSA-ResNet had higher values than that of 2D-ResNet, indicating that 2D-PCSA-ResNet performed better, consistent with our idea in 3.3 again.

### Training and Testing Curves of 2D-Propagation Coefficient Synchronous Adaptive-ResNet

**FIGURE 5:**
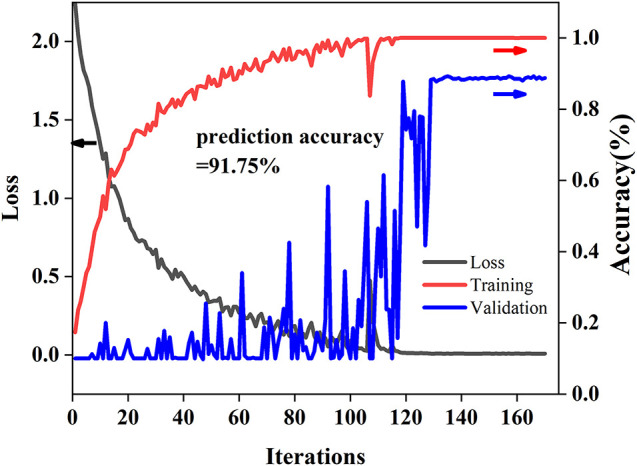
Training and testing curves of 2D-PCSA-ResNet.

Figure 5 shows the training and testing curves of 2D-PCSA-ResNet. As the number of iterations increased, the loss value decreases gradually and the accuracy of the modeling set increased gradually. The accuracy of the validation set fluctuated but finally converged just like the calibration set. The accuracy of the modeling set, validation set, and prediction set was 100, 88.75, and 91.75%, respectively. There was no fitting phenomenon in the 2D-PCSA-ResNet model.

### Feature Clustering Process in 2D-Propagation Coefficient Synchronous Adaptive-ResNet

**FIGURE 6:**
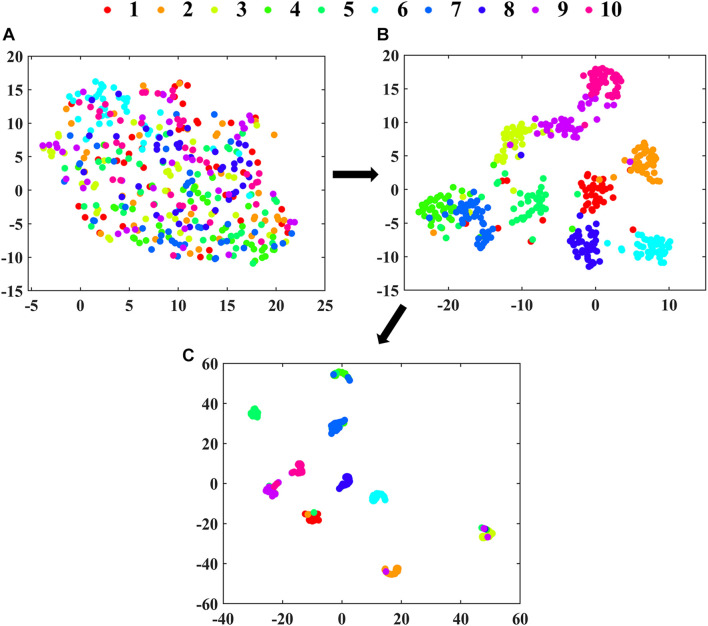
The visualization of clustering effects in layers of **(A)** Max pooling, **(B)** RES. Block4 and **(C)** Dense 10 in 2D-PCSA ResNet by t-SNE.

The clustering effects of features extracted from Max pooling, RES. Block4 and Dense 10 in 2D-PCSA-ResNet are visualized in Figure 6. As the layers went from shallow to deep, the feature clustering phenomenon became more apparent, indicating that the features learned by the deep learning model were more and more representative with the deepening of layers. As shown in Figure 6C, the layer close to the output had successfully learned the soybean seed varieties’ characteristics. Although a small number of data points were misclassified, the classification results were generally satisfactory.

### Saliency Map of the Input Spectral Matrices

**FIGURE 7:**
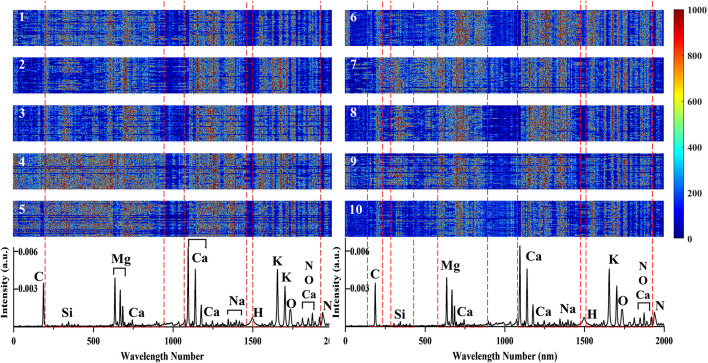
Connected saliency maps for spectral matrix of soybean seeds in prediction set from ten varieties based on 2D-PCSA-ResNet. The two spectra at the bottom are same and are the average spectra from prediction set.

Connected saliency maps of spectral matrices of soybean seeds in the prediction set from ten varieties based on 2D-PCSA-ResNet are shown in Figure 7. The darker the color was, the greater the influence of the corresponding pixels on the discrimination results. (1) It could be seen that the saliency pixels for each variety were distributed in strips. This was because the essence of the input image was spectral matrices. The wavelength and the corresponding intensity of the spectrum could reflect the differences of different samples (Liu et al., 2017). (2) For each variety, there were slight differences in color at the same wavelength. The reason might be that the three spectra that made up the spectral matrix came from three different points on the surface of a soybean seed. And the deep learning model could automatically identify the valuable information for the discrimination (Acquarelli et al., 2017). (3) The saliency maps from different soybean varieties were different. For example, saliency maps from variety 4 had significantly more red spots in the number range of 250–1,000 than that of varieties 3 and 2. These differences were the fundamental reason why the 2D-PCSA-ResNet could distinguish different soybean seed varieties. Moreover, these differences had a certain corresponding relationship with the excitation peak of LIBS spectra, indicating that the content and proportion of elements (C, Si, Mg, Ca, Na, H, K, O, N) in soybean seeds played an important role in variety differences.

### Confusion Matrix Analysis

**FIGURE 8:**
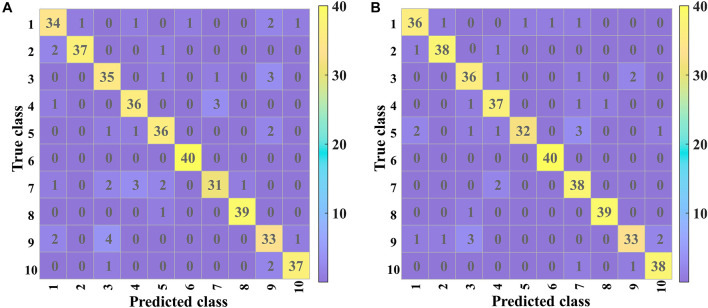
The confusion matrix for the **(A)** validation and **(B)** prediction based on 2D-PCSA-ResNet.

Figure 8 shows the confusion matrices of validation and prediction for 2D-PCSA-ResNet. The details of misclassification of each variety of soybean seeds could be observed clearly.

Good classification performances could be found for Variety 6 (Lvbaoshi) and Variety 8 (Qihuang 34), for which few samples were misclassified. For the validation set, the classification results of Variety 7 (Hedou 25) and Variety 9 (Zhonghuang 13) were poor, more likely to be misclassified as Variety 4 (Jiadou 23) and Variety 3 (Hedou 13), respectively. For the prediction set, Variety 9 (Zhonghuang 13) had similar poor classification results to those in the validation set and Variety 5 (Hedou 33) was more likely to be misclassified as Variety 7 (Hedou 25).

All the samples from Variety 6 (Lvbaoshi) could be correctly classified, which might be attributed to the fact that Lvbaoshi have a distinct color (green) compared to other varieties (yellow), causing the different element distribution from other varieties. Variety 5 (Hedou 33) and Variety 7 (Hedou 25) are easy to be misclassified as each other. This was probably because they were both a type of Hedou with similar genotypes. Variety 9 (Zhonghuang 13) and Variety 3 (Hedou 13) are easy to be misclassified as each other. The reason might be that the saliency maps of Variety 9 and Variety 3 were similar, which was related to the internal structure of the discriminant model. As for variety 9 with the lowest accuracy of 82.5%, special attention should be paid to actual application. Generally, most soybean seeds could be accurately classified, which indicated that LIBS coupled with CNN could be used as a rapid and small-invasive detection method to identify soybean seed varieties.

## Conclusion

Laser-induced breakdown spectroscopy combined with deep learning was successfully applied to the fast identification of soybean seed varieties. It only took 30 s to complete the spectral collection for one soybean seed. Considering the two-dimensional and self-adaptive features of the convolution kernel of CNN, the three spectra of a soybean seed were connected into a spectral matrix as the input. Coupled with spectral matrix, 2D-PSCA-ResNet obtained the highest accuracy in the prediction set with an accuracy of 91.75%. In the future, it can be considered to combine with portable LIBS instruments to realize rapid and on-site identification of soybean seed variety. Meanwhile, more ablation schemes (different laser wavelengths, ablation times, more suitable ablation locations, etc.) can be studied to enhance the detection effects further.

## Data Availability Statement

The raw data supporting the conclusions of this article will be made available by the authors, without undue reservation.

## Author Contributions

XL conceived, designed the experiments and wrote the whole manuscript. XL, ZH, and RC performed the experiments. XL and FL contributed to data analysis, contributed reagents, materials, and analysis tools. All authors contributed to manuscript revision, read, and approved the submitted version.

## Conflict of Interest

The authors declare that the research was conducted in the absence of any commercial or financial relationships that could be construed as a potential conflict of interest.

## Publisher’s Note

All claims expressed in this article are solely those of the authors and do not necessarily represent those of their affiliated organizations, or those of the publisher, the editors and the reviewers. Any product that may be evaluated in this article, or claim that may be made by its manufacturer, is not guaranteed or endorsed by the publisher.
