# Supplementary figures and images for: Fast Identification of Soybean Seed Varieties Using Laser-Induced Breakdown Spectroscopy Combined With Convolutional Neural Network
Source: Front Plant Sci. 2021 Oct 6;12:714557. doi: 10.3389/fpls.2021.714557 (PMC8527016; doi:10.3389/fpls.2021.714557)

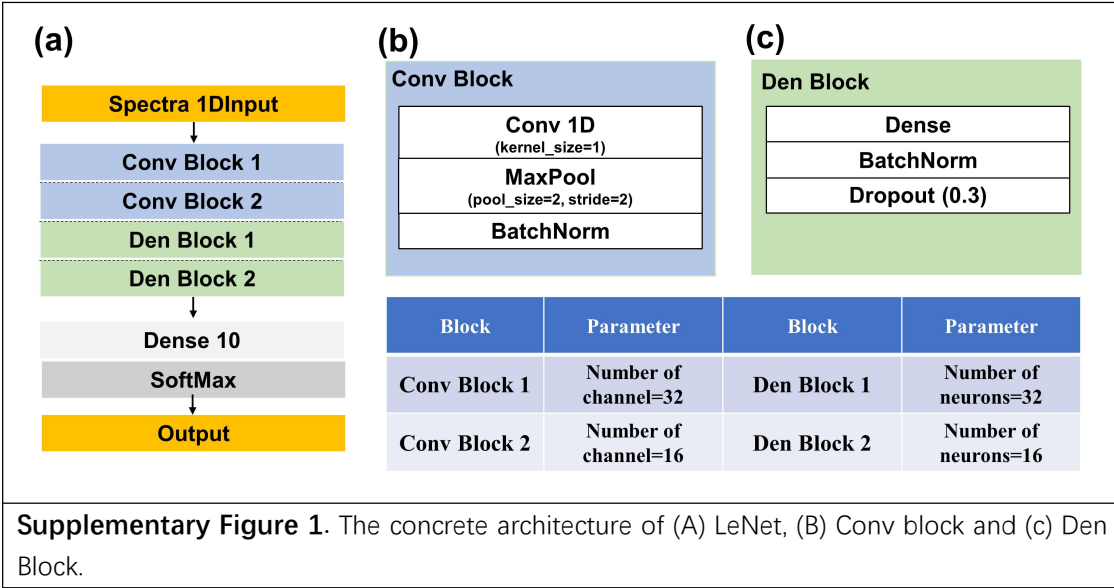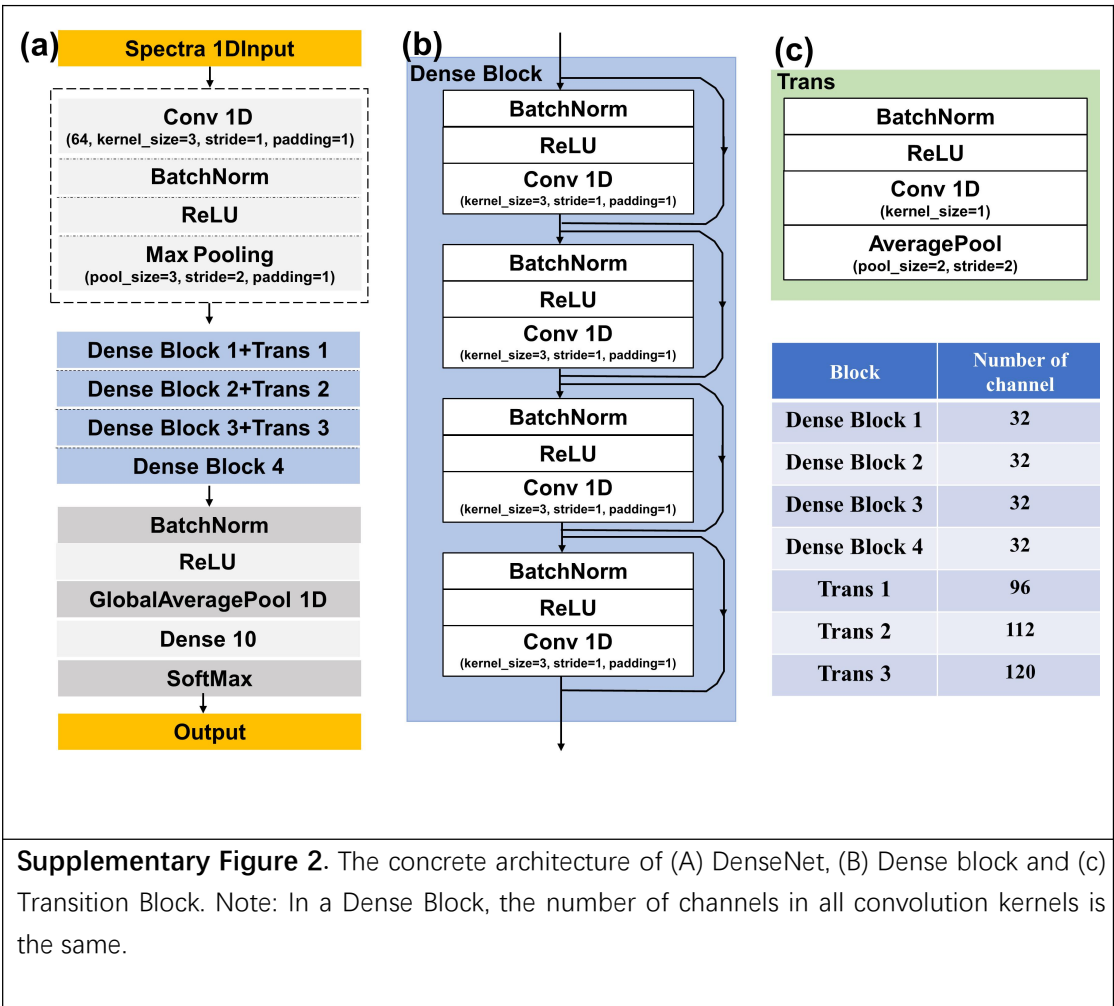

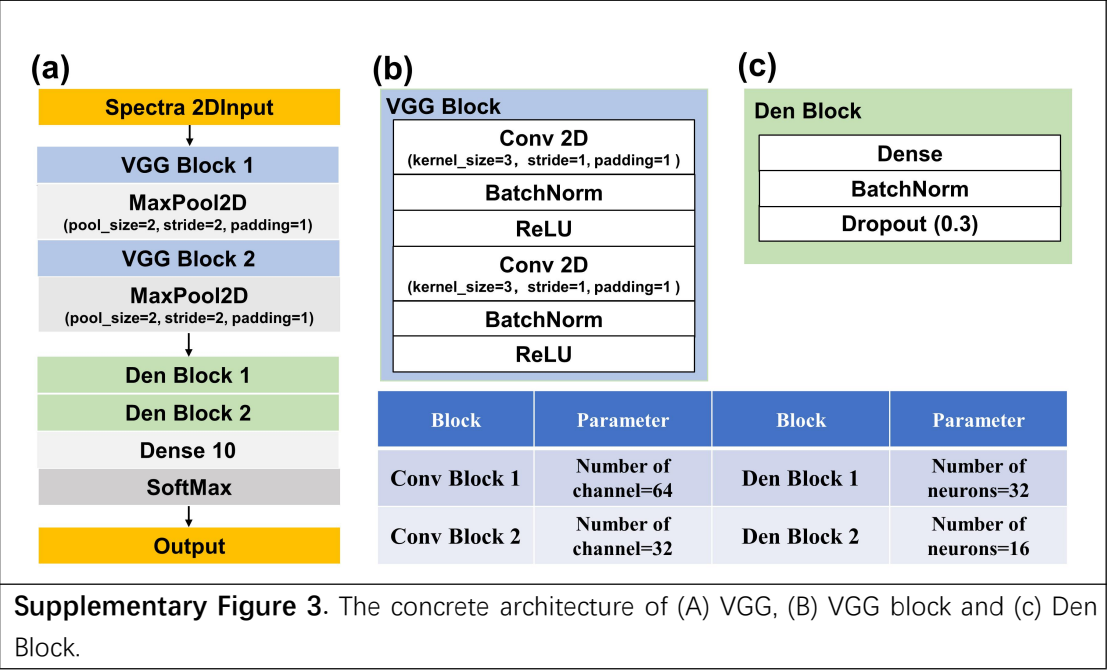

Supplement: Supplementary file 1 [file Data_Sheet_1.pdf]
